# Behavior of *Listeria monocytogenes* and Other Microorganisms in Sliced Riojano Chorizo (Spanish Dry-Cured Sausage) during Storage under Modified Atmospheres

**DOI:** 10.3390/microorganisms9071384

**Published:** 2021-06-25

**Authors:** Elena Gonzalez-Fandos, Maria Vazquez de Castro, Alba Martinez-Laorden, Iratxe Perez-Arnedo

**Affiliations:** CIVA Research Center, Food Technology Department, University of La Rioja, Madre de Dios 53, 26006 Logroño, La Rioja, Spain; mavadeca@gmail.com (M.V.d.C.); alba.mar.lao@outlook.es (A.M.-L.); ipearn@gmail.com (I.P.-A.)

**Keywords:** food safety, chorizo, slicing, MAP, *Listeria monocytogenes*, microbial survival, sausages, dry sausages, RTE, ready-to-eat, LAB, *Micrococcaceae*, yeasts

## Abstract

Sliced ready-to-eat meat products packaged under modified atmospheres are often marketed since they cover consumer demands. The slicing process could be a potential risk for consumers since contamination with *Listeria monocytogenes* could occur during this stage. The current study evaluated the behavior of *L. monocytogenes* and other microorganisms in commercial sliced Riojano chorizo. This meat product was sliced and inoculated with *L. monocytogenes* (3.5 log CFU/g) before packaging under different atmospheres (air, vacuum, 100% N_2_, 20% CO_2_/80% N_2_ and 40% CO_2_/60% N_2_) and stored at 4 °C for up to 60 days. Samples were taken on days 0, 7, 21, 28 and 60 of storage. *L. monocytogenes*, mesophiles, *Enterobacteriaceae*, lactic acid bacteria, *Micrococcaceae*, molds and yeast counts were evaluated. Additionally, water activity, humidity and pH were determined. *L. monocytogenes* counts decreased in inoculated sliced chorizo during storage. Packaging conditions and day of storage influenced microbial counts. After 60 days, a significant reduction (*p* ≤ 0.05) in the initial *Listeria* contamination levels (3.5. log CFU/g) between 1.1 and 1.46 logarithmic units was achieved in the sausages packaged in modified atmosphere. The highest reductions were observed in slices packaged in 40% CO_2_/60% N_2_ after 60 days of storage at 4 °C.

## 1. Introduction

Chorizo is a very popular Spanish dry-cured sausage made of lean pork, pork fat, salt, and spices. More than 30 different types of chorizo have been described based on varying combinations of ingredients and production methods, including drying conditions and the addition or not of starter cultures [1,2]. Riojano chorizo is a traditional meat product from La Rioja, Spain, with Protected Geographical Indication (PGI) status (EC 249/2010) [3]. It is made from lean pork and pork fat added with paprika, garlic and sodium chloride. The chilled meat is minced using an 8–10 mm plate and then mixed with the other ingredients. Starters, sugars and chemical additives as nitrites are usually not added to this type of meat product. The manufacturing process includes low temperatures at the initial stages, usually 4 °C during the first 6 days, and then drying temperatures of 16 °C or below. The process to obtain the final product usually lasts 21 days. Since nitrites and sugars are not added to Riojano chorizo, its characteristics are different from other types of chorizo, as are the factors that affect the growth of microorganisms present in the product. The low processing temperatures, water activity, presence of salt, spices (paprika), and other ingredients (garlic), are the hurdles that could inhibit the growth of foodborne pathogens and spoilage bacteria in this type of chorizo [1]. Few studies have investigated the microbiological safety of this type of chorizo [4].

Several studies have highlighted that pathogenic bacteria such as *Salmonella*, *Staphylococcus aureus*, *Listeria monocytogenes*, *Clostridium perfringens*, *Clostridium botulinum* and *Escherichia coli* can survive in final dry-cured meats and outbreaks can occur [5,6]. During the ripening and drying process, a reduction in pathogen levels has been reported depending on the product processing conditions and composition [6]. Nitrites and nitrates are usually added to raw meat used to prepare dry sausages to reduce the risk of *C. botuliunum* and other pathogens. If nitrite is not added, then the temperature should be below 5 °C until water activity drops below 0.96 to prevent the growth of *C. botuliunum* and botulinum toxin formation in the cases where it is contaminated with *C. botulinum* [6]. *C*. *perfringens* can grow if high processing temperatures are used that allow its multiplication, reaching 6–7 Log CFU/g. *S. aureus* has been found in some dry fermented sausages [7]; this bacterium can grow and produce enterotoxins during the initial stages of sausage fermentation if temperature is high, above 20 °C [2,6]. *Salmonella* spp. may grow in the early stages of processing if temperature is above 20 °C. The addition of nitrites can inhibit the growth of this pathogen in this stage [6]. The abovementioned effect of nitrites on *Salmonella* spp. can be achieved by lowering processing temperature to below 18 °C [6]. As in Riojano chorizo, with processing temperatures of 4 °C during the first 6 days and then 16 °C or lower, growth of *S. aureus* or *Salmonella* spp. or the formation of botulinum toxin are not expected.

*Listeria monocytogenes* causes listeriosis, a severe foodborne disease with a high fatality rate, 17.6% in the European Union in 2019, higher than in 2017 and 2018 (15.6% and 13.6%, respectively) and a high hospitalization rate (92.1% in 2019) [8]. In 2019, 2621 cases were reported in the European Union, of which 225 cases were reported by Spain [8]. The number of cases in 2017 and 2018 were 2479 and 2549, respectively [9]. Clinical listeriosis mainly affects high-risk groups: pregnant women, newborns, elderly and immunocompromised people. It is responsible for abortion, meningitis or septicemic syndrome [10].

*Listeria monocytogenes* is a pathogen of concern in the meat industry because it can be present in the environment and due to its ability to survive in adverse conditions [11]. *L. monocytogenes* can grow in a wide range of pH (4.4–9.6), water activity (0.90–0.99) and temperature (0–45 °C) conditions and in environments containing high concentrations of CO_2_ [11,12]. This pathogen can also form biofilms, and can therefore persist in processing environments for long periods of time [11]. Ready-to-eat meat products, including dry-cured sausages such as chorizo, are often inoculated with *L. monocytogenes* [7,13,14,15,16]. The hazard of the presence of this bacterium tends to decrease during fermentation and drying [17,18]. However, post-processing contamination during slicing has been reported [19].

Prevalence levels of *L. monocytogenes* between 15.8% and 44% have been reported in dry-cured sausages [19,20,21,22]. Moreover, outbreaks of listeriosis have been associated with the consumption of ready-to-eat meat products [23,24]. According to the European Commission Regulation (EC) No 2073/2005, levels of *L. monocytogenes* of less than 2 log CFU/g are tolerated in RTE foods that do not support the growth of this pathogen (RTE products with a pH ≤ 4.4 or a_w_ ≤ 0.92 or pH ≤ 5.0 and a_w_ ≤ 0.94), while zero tolerance is considered in foods that support the growth and have extended shelf life [25].

The main indigenous microorganisms that can play a role and are present in dry-cured sausages are lactic acid bacteria and *Micrococcaceae* [26]. Yeasts have also been highlighted as relevant in some products [27]. These microorganisms are present in raw pork meat and grow during fermentation and drying processes [26]. Moreover, these microorganisms can be effective in controlling the growth of undesirable microorganisms [28,29]. On the other hand, mesophiles, *Enterobacteriaceae* and *E. coli* are useful as indicators of safety in foods [1,2].

Presently, chorizo is often marketed sliced in individual packages under modified atmospheres and stored at refrigeration temperatures. The slicing process could be a potential risk for consumers, since contamination with *L. monocytogenes* could occur during slicing and packaging [20,30,31]. Some authors have reported the capacity of this bacterium to transfer from equipment to dry-cured sausages, including chorizo [18,30,32]. Furthermore, some listeriosis outbreaks have been attributed to cross-contamination during slicing [30,31,33]. Therefore, the food safety risks are higher in sliced products than in traditional products. There is also great concern regarding the microbiological safety of food packaged under modified atmospheres, since *L. monocytogenes* can grow under these conditions [34].

The aim of the present study was to evaluate the survival of *L monocytogenes*, as well as the evolution of other microorganisms (mesophiles, lactic acid bacteria, *Micrococcacceaee*, yeasts, molds and *Enterobacteriaceae*) in sliced Riojano chorizo packaged under modified atmospheres at 4 °C.

## 2. Materials and Methods

### 2.1. Riojano Chorizo Samples

Dry-cured chorizo at the end of the ripening process was provided by a commercial producer. It was aseptically sliced at the processing plant (approximately 2 cm thick) and transported to the laboratory under refrigeration. Upon arrival the chorizo slices were analyzed to guarantee their microbiological quality and physicochemical characteristics and processed on the same day. The following determinations were carried out: *L. monocytogenes*, mesophiles, *Enterobacteriaceae*, Lactic acid bacteria, *Micrococcaceae*, *S. aureus*, sulfite-reducing clostridia, molds and yeast. Additionally, water activity, humidity and pH were determined.

### 2.2. Experimental Design

Two groups of sliced Riojano chorizo were prepared. The first group was inoculated with *L. monocytogenes*, packaged under different conditions and stored at 4 °C. The second group was not inoculated with the pathogen. This second group was packaged and stored under the same conditions as the first group. This group was used to ensure that *L. monocytogenes* inoculation did not influence the evolution of other microbial groups during storage. Ten batches of samples were prepared according to contamination with the pathogen or not and atmosphere packaging conditions. All the batches were stored at 4 °C for up to 60 days. The experimental design included two factors (inoculated with L. monocytogenes and no inoculated) with five levels (packaged in air, packaged under vacuum, packaged in 100% N_2_, packaged in 20% CO_2_/80% N_2_, packaged in 40% CO_2_/60% N_2_).

### 2.3. L. monocytogenes Strains and Inoculum Preparation

Three strains of *L. monocytogenes*: CECT 932 (serotype 1/2a) CECT 934 (serovar 4a) and CECT 4032 (serovar 4b) were used. The strains were selected taking in account the serotypes most often found in meat processing plants and those most often involved in listeriosis outbreaks [12,35,36]. Each strain was individually grown in Brain Heart infusion broth (Oxoid, Hampshire, UK) at 30 °C for 18 to obtain a concentration of 9 log CFU/g. This concentration was verified by serial dilutions and plating on Palcam agar (Oxoid) incubated at 37 °C for 48 h. Serial dilutions in sterile 0.1% peptone solution (Merck, Darmstadt, Germany) (pH 6.2) were performed to obtain the same amount of each strain. A suspension of the three strains of the pathogen was prepared by mixing equal amounts of all three strains to reach a level of approximately 3–3.5 log CFU/g in inoculated chorizo. The prepared suspension was immediately used to contaminate the Riojano chorizo slices.

### 2.4. Chorizo Contamination

Slices from group 1 were inoculated with *L. monocytogenes* as follows: 0.1 mL of the inoculum prepared was dropped onto the slice surface, and spread using a sterile plastic spreader. The inoculated slices were kept at the university pilot plant (12 °C) for 15 min to allow bacteria to attach to the surface. Then, the inoculated slices were introduced in a plastic bag facing the inside of the package and packaged under different atmosphere conditions.

### 2.5. Packaging, Storage Conditions and Sampling

Five slices of chorizo were introduced aseptically in a plastic bag (Dixie, Switzerland) and packaged under different conditions: air, vacuum, 100% N_2_, 20% CO_2_/80% N_2_ and 40% CO_2_/60% N_2_, and stored at 4 °C for up 60 days. Since the shelf life established by the chorizo producer was 60 days (from the end of the ripening process) at 4 °C, the sausages were stored at this temperature until day 60.

The packaging machine used was a Vaessen–Schoemaker (Vaessen–Schoemaker, Spain). The film used had a CO_2_ permeability of less than 13 cm^3^/m^2^/24 h/atm, an O_2_ permeability of less than 5 cm^3^/m^2^/24 h/atm at 25 °C, and a water vapor transmission rate of less than 1.8 g/m^2^/24 h.

Samples were taken on day 0 (after inoculation), and on days 7, 21, 28 and 60 of storage. Three replicates and two determinations in each replicate were carried out for each condition tested. *L. monocytogenes*, mesophiles, *Enterobacteriaceae*, Lactic acid bacteria, *Micrococcaceae*, molds and yeast counts were evaluated. Additionally, water activity, humidity and pH were determined.

### 2.6. Physicochemical Analyses

For water activity, samples were taken from the inner part of the slices and placed in an Aqualab sample cup. Water activity was measured using Aqualab TM 2000 water activity meter (Aqualab, Madrid, Spain). Moisture was determined by drying two homogeneous samples (5 g) at 100 °C to a constant weight. The pH values were determined in a homogenate made of 10 g sample and 90 mL of distilled water using a pH meter model 2002 (Crison Instruments, Barcelona, Spain).

### 2.7. Microbiological Analyses

Twenty-five grams of chorizo were aseptically weighed and homogenized in a Stomacher (IUL, Barcelona, Spain) for 2 min with 225 mL of sterile peptone water (0.1% *w*/*v*) containing tween 80 (1% *w*/*v*) (Oxoid, Basingstoke, UK). Tween 80 added as an emulsifier in the diluent was not observed to be inhibitory to microorganisms in this study as has been confirmed for tween in other studies [2,37]. Further decimal dilutions were made with sterile peptone water (0.1% *w*/*v*). Mesophiles were determined on Plate Count Agar (PCA, Oxoid) following the pour plate method, and incubated at 30 °C ± 1 °C for 72 h. Lactic acid bacteria were determined in MRS agar (Oxoid) incubated at 30 °C ± 1 °C for 72 h. *Micrococcaceae* were determined on Mannitol Salt agar (Oxoid) incubated at 37 °C ± 1 °C for 48 h. To differentiate beneficial *Micrococcaceae* from pathogenic *S. aureus*, 10 colonies per plate were selected and subjected to the determination of coagulase production using rabbit plasma fibrinogen (Oxoid). For yeast and mold counts, Oxytetracycline Glucose Yeast Extract Agar (OGYE) was used (Oxoid). Plates were incubated at 25 °C ± 1 °C for 3–5 days [2]. *Enterobacteriaceae* were determined on plates of Violet Red Bile Glucose agar (Oxoid), the plates being overlaid before incubation at 37 °C ± 1 °C for 18 to 24 h [38]. *Staphylococus aureus* was determined on Baird Park agar containing egg yolk tellurite (Oxoid) incubated at 37 °C ± 1 °C for 48 h. Suspected colonies were tested for coagulase production. Sulfite-reducing clostridia were determined using sulfite polymyxin sulphadiazine agar (Oxoid) incubated at 37 °C ± 1 °C for 24 h under anaerobic conditions. Results were expressed as log10 CFU/g.

For the enumeration of *L. monocytogenes*, 25 grams of chorizo were taken aseptically and homogenized with 225 mL of half Fraser broth (Oxoid). Serial decimal dilutions were performed in sterile peptone water (0.1% *w*/*v*), and 0.1 mL samples of appropriate dilutions were spread onto Palcam agar plates (Oxoid) and incubated at 37 °C ± 1 °C for 48 h. For confirmation, five suspected colonies were isolated and the following identification tests were carried out: Gram stain, oxidase test, catalase reaction, umbrella motility, tumbling motility at 20–25 °C, CAMP test and API Listeria test (BioMérieux, Marcy l’Etoile, France) [39].

Presence/absence of *L. monocytogenes* in 25 g was determined according to the following procedure: after pre-enrichment in half Fraser broth at 30 °C ± 1 °C for 24 h, 0.1 mL were transferred to Fraser broth and incubated at 37 °C ± 1 °C for 48 h, then cultures were plated onto Palcam agar and incubated at 37 °C ± 1 °C for 24 to 48 h. For confirmation, suspected colonies were isolated and identified as described above.

### 2.8. Statistical Analysis

Analysis of variance was performed using the SYSTAT program for Windows; Statistics v. 5.0 (Evanston, Illinois, 1992). Tukey’s test for comparison of means was performed using the same program. Plate count data were converted to logarithms prior to their statistical processing. There were six replicates per treatment and storage time. Significance level was defined at *p* ≤ 0.05.

## 3. Results

**Figure 1:**
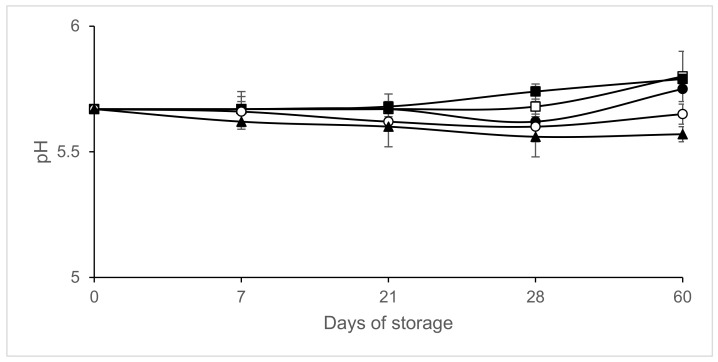
pH evolution in inoculated sliced chorizo packaged in modified atmospheres. Packaging conditions: Air (□), 100% Nitrogen (■), Vacuum (●), 20% CO_2_/80% N_2_ (○), 40% CO_2_/60% N_2_ (▲).

The water activity of the chorizo before packaging was 0.848 ± 0.001, pH was 5.67 ± 0.01 and humidity 24.22 ± 0.50%. After 7 days of storage, the pH was slightly lower in the slices packaged in atmospheres containing CO_2_ than in those packaged under vacuum or 100% N_2_ (Figure 1). No significant differences (*p* > 0.05) in pH were found among the chorizo samples packaged under different atmospheres. Water activity remained relatively constant at values of around 0.847, except on day 60 of storage in slices packaged in air with values of 0.840 ± 0.000. Humidity did not decrease significantly throughout storage, remaining at around 24%, except in the slices packaged in air with humidity values of 20.35% on day 60 of storage. No significant differences (*p* < 0.05) in pH, water activity or humidity were found between the samples inoculated with *L. monocytogenes* and those not inoculated (data not shown).

Neither *S. aureus* nor sulfite-reducing clostridia were detected in sliced samples provided by the producer. Mesophile, lactic acid bacteria, *Micrococacceaea* and yeast counts before contamination were 7.32 ± 0.15 log CFU/g, 6.88 ± 0.03, 5.95 ± 0.27 and 3.63 ± 0.07, respectively. *Enterobacteriaceae* and molds were not detected in any samples before contamination. No significant differences (*p* < 0.05) were observed in these microbial groups between samples taken before contamination and after contamination and packaging (day 0).

**Figure 2:**
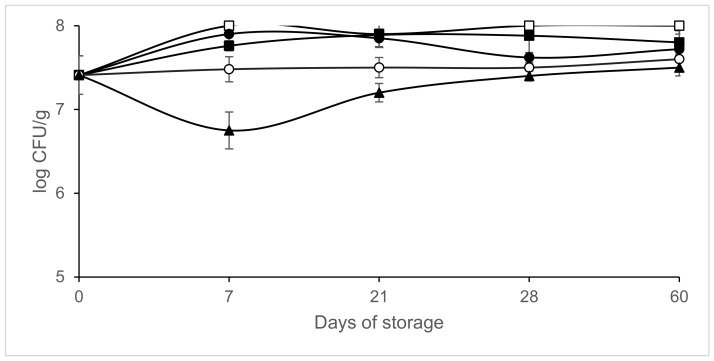
Effect of packaging conditions on mesophile counts in inoculated sliced chorizo packaged in modified atmospheres. Packaging conditions: Air (□), 100% Nitrogen (■), Vacuum (●), 20% CO_2_/80%N _2_ (○), 40% CO_2_/60% N_2_ (▲).

**Figure 3:**
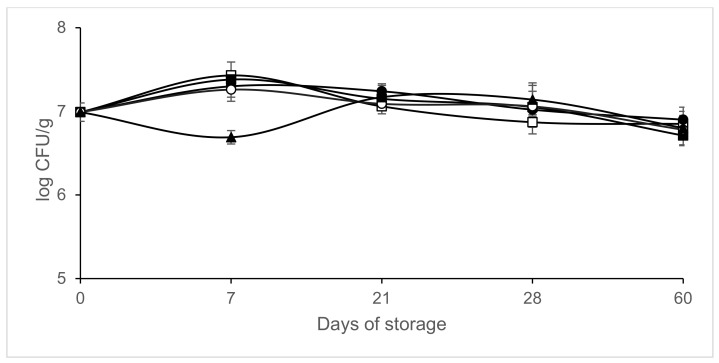
Effect of packaging conditions on lactic acid bacteria counts in inoculated sliced chorizo packaged in modified atmospheres. Packaging conditions: Air (□), 100% Nitrogen (■), Vacuum (●), 20% CO_2_/80% N_2_ (○), 40% CO_2_/60% N_2_ (▲).

**Figure 4:**
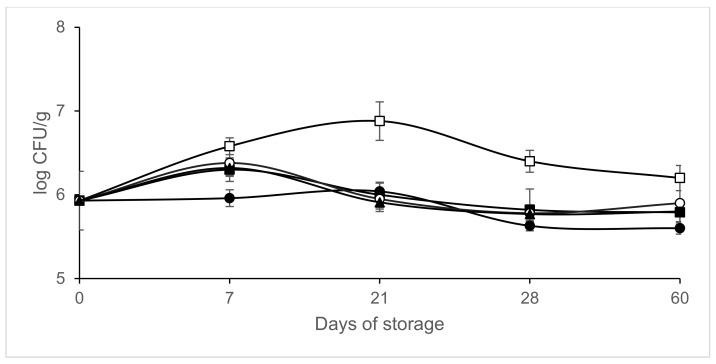
Effect of packaging conditions on *Micrococcaceae* counts in inoculated sliced chorizo packaged in modified atmospheres. Packaging conditions: Air (□), 100% Nitrogen (■), Vacuum (●), 20% CO_2/_80% N_2_ (○), 40% CO_2/_60% N_2_ (▲).

**Figure 5:**
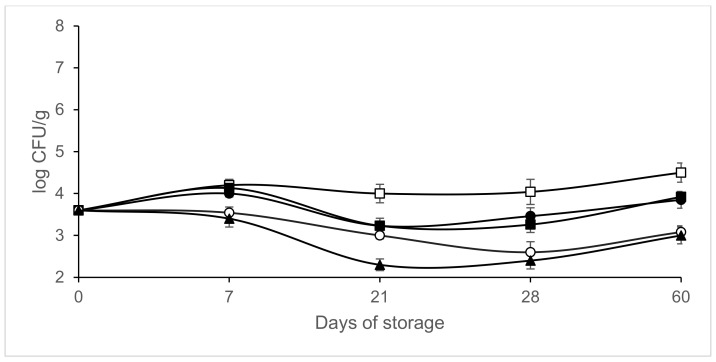
Effect of packaging conditions on yeast counts in inoculated sliced chorizo packaged in modified atmospheres. Packaging conditions: Air (□), 100% Nitrogen (■), Vacuum (●), 20% CO_2/_80% N_2_ (○), 40% CO_2_/60% N_2_ (▲).

Figure 2, Figure 3, Figure 4 and Figure 5 shows the evolution of the different microbial groups in inoculated slices throughout storage. Initial mesophile counts were 7.41 ± 0.23 log CFU/g. A decrease in mesophile counts was observed in slices packaged in 40% CO_2/_60% N_2_ on day 7 of storage. Significantly lower (*p* ≤ 0.05) mesophile counts were observed in slices packaged in atmospheres containing CO_2_ than in those packaged in air (Figure 2). No significant differences (*p* > 0.05) were found in mesophile counts between samples packaged in air and those packaged in atmospheres containing 100% N_2_ throughout storage at 4 °C. Final mesophile counts ranged between 7.5 and 8 log CFU/g (day 60). High initial lactic acid bacteria counts around 7 log CFU/g were found. Lactic acid bacteria increased slightly on day 7, except in the samples packaged in 40% CO_2_/60% N_2_. After day 7, the lactic acid bacteria populations remained stable, with populations similar to the initial ones after 60 days of storage (Figure 3). Final lactic acid bacteria counts ranged between 6.71 and 6.9 8 log CFU/g (day 60). Initial *Micrococcaceae* counts were 5.93 ± 0.35 log CFU/g. *Micrococcaceae* populations increased in air-packaged slices until day 21. In the samples packaged in modified atmospheres, a slight initial increase was observed except in the vacuum packaged sausages; later the populations stabilized, being similar to the initial counts (Figure 4). Final *Micrococcaceae* counts ranged between 5.6 and 6.2 log CFU/g (day 60). Yeast counts were lower in slices packaged in atmospheres containing CO_2_ than in those packaged under vacuum or 100% nitrogen. The greatest increase in yeast counts occurred in samples packaged in air (Figure 5). Final yeast counts ranged between 3 and 4.5 log CFU/g (day 60). *Enterobacteriaceae* remained below the detection limit (<1 log CFU/g). Molds were not detected, remaining under the detection limit (<2 log CFU/g). No significant differences (*p* < 0.05) in lactic acid bacteria, *Micrococcaceae* or yeast and molds were found between the samples inoculated with *L. monocytogenes* and those not inoculated (data not shown).

**Figure 6:**
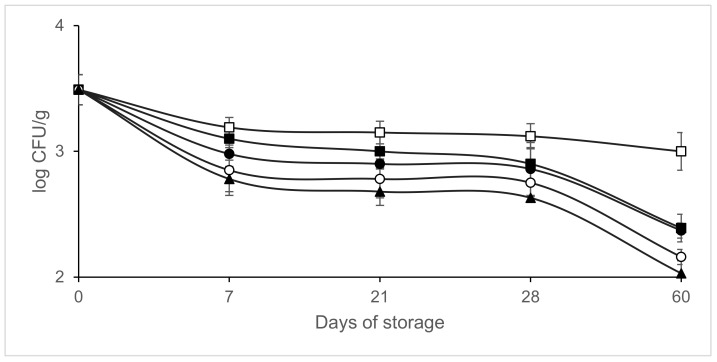
Effect of packaging conditions on the survival of *Listeria monocytogenes* on inoculated sliced chorizo packaged in modified atmospheres. Packaging conditions: Air (□), 100% Nitrogen (■), Vacuum (●), 20% CO_2_/80% N_2_ (○), 40% CO_2/_60% N_2_ (▲).

*Listeria monocytogenes* was not detected in the non-inoculated slices. After inoculation, *Listeria monocytogenes* counts were 3.49 ± 0.12 log CFU/g. Figure 6 shows the evolution of *L. monocytogenes* in packaged sausages under different conditions throughout storage at 4 °C. Growth of *L. monocytogenes* was not observed under any of the conditions studied. After 60 days, a significant reduction (*p* ≤ 0.05) between 1.1 and 1.46 logarithmic units of the *Listeria* populations was achieved in the sausages packaged in modified atmosphere compared to the initial counts (day 0), while in those packaged in air the decrease was 0.49 logarithmic units. The greatest reduction was observed in sausages packaged in 40% CO _2_/ 60% N_2_ with 1.46 log unit reductions. Significant reductions (*p* ≤ 0.05) in this pathogen were found between samples packaged in atmospheres containing CO_2_ and those packaged in air throughout storage. On day 7 of storage, the slices packaged in 40% CO_2/_60% N_2_ showed reductions of *L. monocytogenes* of 0.41 log units compared to those packaged in air. On day 60 of storage, the slices packaged in 40% CO_2/_60% N_2_ showed reductions of *L. monocytogenes* of 0.97 log units compared to those packaged in air.

## 4. Discussion

The pH of Riojano chorizo at the end of the ripening process (final product) was higher (5.67) than in other kind of chorizos [13,40,41,42,43,44]. Encinas et al. [13] reported pH values between 5.10 and 5.19 in chorizos without starter and between 4.29–4.57 when starter culture was added. Chorizo pH values between 4.7 and 5.4 have been reported by Thevenot et al. [44], whereas Menendez et al. [45] described pH values of 5.12 ± 0.28. Therefore, these pH values can be considered to be a product characterizing value in Riojano chorizo. Similar pH has been reported in other dry fermented sausages in which sugar was not added [7,46]. Other traditional chorizos are prepared without adding starters, but adding sugars, resulting in a lower pH of the final product (around 5.1–5.3) [47,48]. Similar values of pH (5.42–5.55) and a_w_ (0.826–0.86) have been reported in salami by Tirloni et al. [49]. As the pH is relatively high, the drying process is critical to achieve a low a_w_. Thus, in Riojano chorizo a_w_ is the main limiting factor for pathogen growth and survival [50]. In the present study, no changes in a_w_ were observed in slices packaged in vacuum. These results coincide with those reported by Tirloni et al. [49] and Byeslahov et al. [51].

The main indigenous bacteria involved in the processing of dry-cured sausages are lactic acid bacteria, although depending on the type of product *Micrococcaceae* may also play an important role [26,52]. Lactic acid bacteria are relevant since they play a role in the fermentation process and preservation of sausages [53,54]. *Micrococcaceae* produce proteases and lipases and can reduce nitrates, being involved in aroma and color development [52]. Moreover, some studies have pointed out that *Micrococcaceae* can play a role in controlling pathogens in dry fermented sausages, including *L. monocytogenes* [55,56]. High lactic acid bacteria and *Micrococcaceae* counts at the end of the ripening stage have been also reported by other authors [45,50,57]. In the present study, lactic acid bacteria and *Micrococaceae* were the predominant bacteria. Similar levels of lactic acid bacteria (6–8 log CFU/g) and *Microcacacceae* (6–7 log CFU/g) have been reported in other dry fermented sausages processed without addition of starter [45,50,57]. On the other hand, Cardinali et al. [58] observed that *Micrococacceae* were mainly isolated in dry fermented sausages in which nitrites or nitrates were not added. Similar mesophilic counts have been reported by Tirloni et al. [49] in salami, corresponding mainly to lactic acid bacteria. Lower initial mesophiles counts in chorizo packaged under vacuum were reported by Cava et al. [35] (about 5.5 log CFU/g), although behavior was similar since a slight increase was observed on day 30 of storage, and afterwards the population remained constant. Cava et al. [35] did not analyze lactic acid bacteria, since lower mesophile counts were observed, and it is also assumed that lower lactic acid bacteria were present in the chorizo samples. Higher mesophile counts in chorizo have been found by Menendez et al. [42] (8.79 log CFU/g). As in the present study, Tirloni et al. [49] did not observe significant changes in mesophile and lactic acid bacteria counts in vacuum-packed salami.

Cava et al. [35] reported similar yeast counts in chorizo at the end of the ripening stage, at around 3.5 log CFU/g. However, after 30 days of vacuum storage these authors observed a reduction of yeast counts to values of 3 log CFU/g, the reduction being higher after 60 days of storage. Yeast levels in the final product of around 2–4 log CFU/g have been reported in other traditional dry sausages processed without addition of starter culture [7,46,50]. The presence of yeasts in different types of chorizo suggests their role in the process [27]. *Debaryomyces hansenii* has been reported as the dominant yeast found in chorizo [27].

*Enterobacteriaceae* counts were below the detection limit. Similar results have been reported by Tirloni et al. [49]. However, Menendez et al. [42] detected *Enterobacteriaceae* in one third of the chorizo analyzed, with counts of 2.35 log CFU/g. Low *Enterobacteriaceae* counts are associated with good hygiene conditions [59]. As in the present study, other authors did not isolate *S aureus* or sulfite-reducing bacteria in dry-cured sausages at the end of the ripening process (final product) [59].

As expected, no growth of *L. monocytogenes* was observed in any of the conditions studied since water activity was below 0.85, and the limit value for growth of this pathogen is 0.90 [36]. However, this bacterium can still survive under these conditions [60]. It should be noted that this pathogen is able to survive in dry fermented meat products due to its high tolerance to factors such as low pH or high salt levels [14]. Higher reductions of *L. monocytogenes* in sliced chorizo packaged in vacuum conditions and 20% CO_2_/80% N_2_ on day 7 of storage at 3 °C (2.7 and 2.31 log units, respectively) and reductions of 2.70 log units after 30 days of storage have been reported by Menendez et al. [45]. In our study, after 28 days of storage at 4 °C reductions of 0.63 and 0.74 log units were observed in sliced chorizo packaged in vacuum and 20% CO_2_/80% N_2_, respectively. These differences can be explained by the characteristics of the chorizo studied. *L. monocytogenes* survival in dry-cured sausages is affected by pH, low a_w_ and lactic acid bacteria [17,61,62,63]. In the present study, the initial a_w_ was 0.848 ± 0.001 and the pH 5.67 ± 0.01. In contrast, Menendez et al. [45] reported slightly higher a_w_ (0.875 ± 0.00) and lower initial pH (4.99 ± 0.01). However, no reductions in *L monocytogenes* in sliced chorizo inoculated with 3 log CFU/g and packaged in vacuum after 30 days of storage at 4 °C have been reported by Cava et al. [35]. Nevertheless, after 60 days of storage these authors observed the presence of this pathogen, but the counts were below 1 log CFU/g. In contrast, in our study reductions of 0.63 and 1.12 log units were observed in slices packaged under vacuum after 28 and 60 days of storage, respectively. In the study carried out by Cava et al. [35], the pH of the chorizos was slightly lower than that reported here, at 5.50 ± 0.04 (5.67 ± 0.01 in the present study) and a_w_ levels of 0.80 ± 0.01, lower than reported here (0.848 ± 0.001). Higher reductions (1.5 log CFU/cm^2^) of *L monocytogenes* counts in pepperoni packaged under vacuum after 4 days of storage at 4 °C, and under the detection limit after day 60 of storage have been reported by Byelashov et al. [51]. These higher reductions can be explained by the lower pH (4.67 ± 0.09) and the lower a_w_ (0.827 ± 0.01). As highlighted by other authors, low water activity in dried sausages is one important hurdle for controlling pathogens such as *L. monocytogenes* [12,44,48]. According to Christieans et al. [61], nitrite is an important hurdle for controlling *L. monoccytogenes* in the fermentation stage of dry fermented sausages. Additionally, Possas et al. [18] highlighted the role of nitrite in controlling this pathogen in sliced chorizo. It is important to mention that nitrites added to the sausage mixture at the beginning of the process decreased rapidly, and the residual nitrite amounts found in the final product are usually below 10 ppm [56,61]. Paprika and garlic used in the preparation of Riojano chorizo contain nitrates, phenolic compounds and other bioactive substances with potential antioxidant and antimicrobial properties [64]. Consequently, some amounts of nitrate can be present in the sausage mixture when paprika and garlic are added. Moreover, some authors have not reported the nitrite levels in the final product, only the amount added at the beginning of the process. Since this study and those carried out by other authors examined the survival of *L. monocytogenes* inoculated in the final product after slicing, the amount of nitrites present might be too low (below 10) to be effective against the pathogen studied.

The lactic acid bacteria present in Riojano chorizo (6.69–7.43 log CFU/g) could exert an inhibitory effect on this bacterium [28,29]. According to Al-Zeyara et al. [28] and Cornu et al. [29], when lactic acid bacteria are present in meat products at levels higher than 4.5 log CFU/g, they can inhibit the aforementioned pathogen by originating conditions that are unfavorable for this pathogen. This inhibiting effect has been observed at low temperatures [65]. Moreover, lactic acid bacteria can be good competitors for nutrients [66]. As in the present study, Jacome et al. [54] did not observe any effect on lactic acid bacteria populations between slices of chorizo inoculated with *L. monocytogenes* and uninoculated slices.

The *Micrococcaceae* present in Riojano chorizo (5.60–6.88 log CFU/g) could play a role in controlling *L. monocytogenes* [55].

The antagonist effect of yeasts against pathogens including *L. monocytogenes* has been reported [67,68,69]. Some authors have evaluated the use of *D. hansenii* as a protective culture to control the growth of *L. monocytogenes* in sliced dry-cured ham [70]. However, it has been observed that *D. hansenii* has a limited action on *L. monocytogenes* in slice ham and even promotes the growth of this pathogen [70]. The proteolytic activity of this yeast results in the generation of peptides and free amino acids that could foster the growth of other microorganisms such as *L. monocytogenes* [71,72]. Therefore, these authors concluded that this yeast was not a good option as a protective culture in ham [70].

Other authors have reported lower reductions of *L. monocytogenes* in dried sausages stored at 20 °C than at 4 °C [35,60,73,74]. This advantage of *L. monocytogenes* at low temperatures can be explained by the reduced lactic acid bacteria activity at low temperatures [73]. Moreover, other authors have observed a decrease in the prevalence of *L monocytogenes* in chorizo after storage at 4 °C or 10 °C, being lower at 10 °C than at 4 °C [20]. Some authors suggest maintaining sliced dried sausages at ambient temperature to reduce *L. monocytogenes* [35].

Sliced *dry*-cured sausages are packaged in MAP to increase their shelf life and preserve their quality [75]. However, there is a great concern regarding the behavior of *L. monocytogenes* under these conditions [76]. The results obtained in the present study showed that atmospheres containing CO_2_ were more efficient in reducing of *L. monocytogenes* in sliced Riojano chorizo than under vacuum, 100% N_2_ or air conditions, with greater reductions when CO_2_ levels increased. These findings coincide with those reported by other authors in meat products [77,78]. Most studies carried out in sliced dried sausages, including chorizo, are related to samples packaged under vacuum conditions [18,35,51,52]. The results reported in the present study suggest the advantage of using atmospheres containing CO_2_ to control *L. monocytogenes* since higher reductions were observed under these conditions.

Riojano chorizo is a ready-to-eat meat product with a_w_ ≤ 0.92. The European Commission has established a food safety criterion for this type of foods of 2 log CFU/g of *L. monocytogenes* during product shelf life [25]. In 2019, 2.1% of the RTE meat products exceeded the levels established by Regulation (EC) No 2073/2005 [8,25]. In the present study, *L. monocytogenes* counts exceeded the critical limit of 2 log CFU/g. To obtain safe products preventive measures must be taken to reduce contamination. It must be stressed that the initial inoculum of *L. monocytogenes* was high (3 log CFU/g). The natural contamination of *L monocytogenes* found in dry-cured sausages is usually below 2 log CFU/g, although levels above 2 log CFU/g have also been reported [7,14,15,16].

High prevalence of *L. monocytogenes* has been detected in dry-cured sausages processing plants, including equipment and prepared products [20,79]. Martin et al. [19] carried out a study on the prevalence of this bacterium in Spanish traditional factories producers of fermented sausages. These authors reported the presence of this pathogen in 11.8% of the samples taken from the equipment and 15.8% of the final products. Gomez et al. [20] detected the presence of *L. monocytogenes* in 22.72% of the equipment and surfaces analyzed and in 36.4% of the dry-cured sausages (chorizo and salchichon). Talon et al. [80] detected this bacterium in 6.7% of the surface and equipment from dry sausages processing plants; in some samples this pathogen reached levels of 4 log CFU/cm^2^. Higher prevalence of this bacterium in a meat cutting facility was reported by Zwirztiz et al. [80]. The cross-contamination of sliced chorizo during slicing is a relevant source that increases the prevalence and populations of this bacterium in final product [18]. Possas et al. [18] have estimated that high concentrations of this bacterium could be transferred from the slicing equipment to the slices. *L. monocytogenes* has been detected in meat processing plant surfaces and equipment even after cleaning and disinfection [20]. Consequently, measures should be taken to ensure effective cleaning and disinfection to minimize cross-contamination with this pathogen. The present work only examines the post-process contamination that might occur during slicing of Riojano chorizo and not the process of manufacture.

Riojano chorizo is a low-acid dry sausage, since no starters or sugars are added, with pH levels normally above 5.6 [26]. In contrast, the pH of high acid sausages is lower than 5.3 and can be 4.5. The stability and safety of low-acid sausages depends on water activity, with values being significant at below 0.89, as reported in the present work, and processing temperatures below 15 °C [26]. Since chorizo can be contaminated with *L. monocytogenes* during slicing [18,31] special care should be taken in the cleaning and disinfection of slicing equipment to avoid cross-contamination.

## 5. Conclusions

Sliced Riojano chorizo do not support the growth of *L. monocytogenes*, but this bacterium can survive in this product. Depending on the initial contamination during slicing the decrease could be insufficient and *L. monocytogenes* can exceed the tolerable limit of 2 log CFU/g.

The data obtained suggests that further safety factors should be considered for Riojano chorizo (antimicrobials, starter cultures and/or added sugar) to lower pH to ensure that the products are safe from *L. monocytogenes.* The application of HACCP, Good Manufacturing Practices, and proper cleaning and disinfection measures are essential to prevent contamination with *L monocytogenes* and obtain a safe meat product.

## Data Availability

Research data are not shared.
